# Epithelial-Mesenchymal Transition in Metastatic Cancer Cell Populations Affects Tumor Dormancy in a Simple Mathematical Model

**DOI:** 10.3390/biomedicines2040384

**Published:** 2014-12-09

**Authors:** Adam L. MacLean, Heather A. Harrington, Michael P. H. Stumpf, Marc D. H. Hansen

**Affiliations:** 1 Theoretical Systems Biology, Department of Life Sciences, Imperial College London, Sir Ernst Chain Building, London SW7 2AZ, UK; E-Mails: adam.maclean09@imperial.ac.uk (A.L.M.); Heather.Harrington@maths.ox.ac.uk (H.A.H.); m.stumpf@imperial.ac.uk (M.P.H.S.); 2 Department of Physiology and Developmental Biology, Brigham Young University, Provo, UT 84602, USA

**Keywords:** cancer growth, metastasis, chemotherapy, mathematical modeling

## Abstract

Signaling from the c-Met receptor tyrosine kinase is associated with progression and metastasis of epithelial tumors. c-Met, the receptor for hepatocyte growth factor, triggers epithelial-mesenchymal transition (EMT) of cultured cells, which is thought to drive migration of tumor cells and confer on them critical stem cell properties. Here, we employ mathematical modeling to better understand how EMT affects population dynamics in metastatic tumors. We find that without intervention, micrometastatic tumors reach a steady-state population. While the rates of proliferation, senescence and death only have subtle effects on the steady state, changes in the frequency of EMT dramatically alter population dynamics towards exponential growth. We also find that therapies targeting cell proliferation or cell death are markedly more successful when combined with one that prevents EMT, though such therapies do little when used alone. Stochastic modeling reveals the probability of tumor recurrence from small numbers of residual differentiated tumor cells. EMT events in metastatic tumors provide a plausible mechanism by which clinically detectable tumors can arise from dormant micrometastatic tumors. Modeling the dynamics of this process demonstrates the benefit of a treatment that eradicates tumor cells and reduces the rate of EMT simultaneously.

## 1. Introduction

Cancer metastasis greatly complicates the treatment of solid tumors. Cells derived from tumors primarily use the vascular and lymphatic systems as networks for transport to and colonization of distant sites. The rate at which individual cells detach from tumors and enter circulation can be significant [1], allowing large numbers of tumor cells to be detected in circulation and allowing for the establishment of numerous micrometastatic lesions in distant target tissues. Micrometastases, consisting of only a few tumor cells can remain dormant for extended periods, decades in the case of some tumor types, before suddenly switching from this dormant state and initiating rapid tumor growth [2]. How micrometastases are established and maintained, as well as how they move beyond dormancy, remains unclear.

For many tumor types, cancer stem cells play an important role in successful cancer metastasis [3,4]. Unlike more differentiated tumor cell types, cancer stem cells have the ability to reconstitute an entire tumor from even a single cancer stem cell precursor [5]. Besides this, cancer stem cells also exhibit other critical properties. They tend to be more resistant to chemotherapeutic drugs than bulk cancer cells [6,7]. Cancer stem cells are a tumor cell subpopulation, whose frequency in the total tumor cell population ranges from extremely rare to abundant [8]. The abundance of the cancer stem cell subpopulation in different cancer cell lines explains the variability in the number of injected cancer cells required to successfully establish a tumor *in vivo* [9]. While the dogma has been that stem cells are only repopulated from other stem cells, there is much evidence that calls this idea into question [10,11].

Mesenchymal cells play a similarly important role in cancer metastasis. In tumors of epithelial origin, mesenchymal cells exhibit a dramatically reduced epithelial phenotype. Instead of demonstrating polarity and the cell-cell adhesion typical of epithelial cells, mesenchymal cells are highly invasive, migratory and solitary. Like cancer stem cells, mesenchymal cells also tend to be more drug resistant [12] and occur as a subpopulation of varying proportion within the tumor cell type. There is persuasive evidence that mesenchymal cells act as cancer stem cells in epithelial tumor populations [10,11,13].

Mesenchymal cells are generated when epithelial cells are triggered to detach from the epithelial tissue and initiate invasion and migration as solitary cells, a process termed epithelial-mesenchymal transition (EMT). This process is thought to occur in solid tumors and to play a fundamental role in cancer invasion and metastasis [14,15]. EMT in development and cancer is initiated by a number of cellular signaling pathways, notably by activation of the c-Met receptor tyrosine kinase [16]. c-Met is the receptor for hepatocyte growth factor (HGF)/scatter factor [17]. Its activation drives epithelial scattering *in vitro* [18], and has more recently been linked to cancer stem cell phenotypes [19,20,21] and resistance to apoptosis [22].

The current paradigm suggests that EMT events drive metastasis by producing mesenchymal cells that escape the primary tumor and migrate to distant sites, whereby they can revert to an epithelial state. However, the connection between EMT and cancer stem cells might have important implications beyond dissemination and colonization of metastatic disease. It has been postulated that EMT events might contribute to later events in metastasis, including dormancy [23]. However, exploring how EMT events might contribute to the dormancy of tumors has proven difficult to address experimentally [24].

Mathematical models of tumor cell populations with a stem cell subpopulation have been successfully used to understand how stem cell populations affect cancer biology in both solid tumors and leukemias [25,26,27,28,29]. In this work, we analyze how the rates of EMT, proliferation, senescence and cell death affect population dynamics in a model for dormant tumors. Our goal is to define how changes in each of these cellular properties affects the maintenance of a dormant population dynamic. We use a highly generalized, abstract model in the hope that some broadly applicable hypotheses about dormancy emerge.

In our chosen model for micrometastatic tumors, a progenitor cancer stem cell that has already colonized a distant tissue divides asymmetrically to generate one stem and one differentiated epithelial progenitor cell. The differentiated epithelial progenitor cell can divide some number of times before becoming senescent; we do not explicitly model senescence, since we consider senescent cells inert from a tumor progression standpoint. In this simple model for tumor population dynamics, tumors arising from single colonizing progenitor cells rapidly reach a stable equilibrium with small numbers of cells, reminiscent of a dormant micrometastatic tumor that would result from balanced proliferation and cell death [30]. Most of the tumor cells in these micrometastatic lesions are epithelial, with very few mesenchymal cells. We combine stochastic and deterministic approaches to show how alterations in apoptosis, proliferation and the occurrence of EMT influences the behavior of these “dormant” micrometastatic tumors. Variations in the rate of EMT dramatically alter the behavior of the metastatic tumor population, much more so than alterations in the rates of apoptosis, proliferation or capacity for multiple rounds of cell division, suggesting that EMT events can have a strong effect on the maintenance of a dormant state. Finally, we also model the chemotherapeutic treatment and recovery of these tumors, including under conditions where EMT is prevented. The implications of these findings are discussed.

## 2. Results

### 2.1. Deterministic Population Dynamics of Dormant Metastatic Tumors

Micrometastatic tumors can remain dormant, and undetectable, for significantly extended periods before suddenly entering a growth phase that gives rise to large, rapidly growing tumors. How tumors remain and exit dormancy remains poorly understood. While the role of cells with cancer stem cell properties in maintaining micrometastatic tumors is accepted, the implications of generating cancer stem cells from EMT events has not been carefully studied in the context of tumor dormancy. We sought to understand the population dynamics of metastatic tumors that arise from a single colonizing, stem cell property-bearing cell using deterministic models. Ordinary differential equation (ODE) models enable the study of the dynamical behavior of cellular species, such as cancer cell populations, and have been used extensively to this end [31]. The recent understanding of the relationship between mesenchymal cells or EMT and cancer progression has only been addressed using a modeling approach in a few pioneering studies [32,33], the most notable of which used mathematical modeling to study whether EMT or rapid expansion of stem cell populations better accounted for the ability of breast cancer cells to form mammospheres in culture. To our knowledge, no model includes EMT as it relates to the cancer stem cell paradigm in the context of metastatic tumor dormancy.

**Figure 1:**
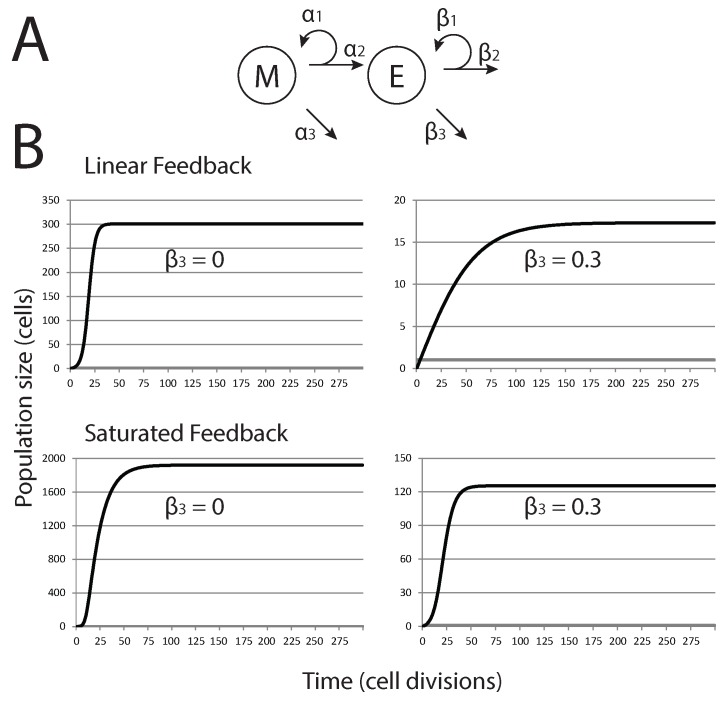
Tumor population dynamics in models of cancers derived from pioneering stem cells. (**A**) Diagrammatic description of the basic model with *M* (mesenchymal cells), *E* (epithelial cells) and their rate parameters; (**B**) trajectories for *M* (grey lines) and *E* (black lines), for the two model variations and two different values of $\beta_{3}$ (senescence of *E*).

Here, we develop a simple model that describes the population dynamics of mesenchymal (stem-like) cells, *M*, and epithelial (differentiated) cells, *E*; the schema shown in Figure 1A. We consider two different forms of feedback controlling the growth of epithelial cells. First, a linear feedback model is discussed; then, we introduce saturated feedback as an alternative growth-limiting mechanism. This saturated feedback model is analyzed in detail. Both models are based on those developed by Johnston *et al.* [25], and both models give rise to a highly stable population equilibrium that might represent a dormant metastatic tumor under the balanced proliferation model [30].

In the case of linear feedback, senescence of epithelial cells is determined by a factor (the saturation term) that is proportional to the current population size (kE) [25], and we have:

(1) $$\begin{array}{cc} \frac{dM}{dt} & =(\alpha_{1}-\alpha_{2}-\alpha_{3})M \end{array}$$

(2) $$\begin{array}{cc} \frac{dE}{dt} & =\alpha_{2}M+\left(\beta_{1}-\beta_{2}-\beta_{3}-kE\right)E, \end{array}$$

where *M* and *E* are the population sizes of mesenchymal and epithelial cells, respectively, and all of the parameters used are described in Table 1. Equation (4) can be solved analytically,

(3) $$\begin{array}{c} M\left(t\right)=M_{0}e^{\alpha t} \end{array}$$

where $\alpha =\alpha_{1}-\alpha_{2}-\alpha_{3}$ and $M_{0}$ is the initial population size of *M*. This gives simple exponential growth or decay for positive or negative *α*, respectively.

Motivated by the linear feedback model, we next consider a saturated feedback model, where senescence of epithelial cells ($\beta_{2}$) is governed by the epithelial population size, but with a maximum per-capita growth rate limitation that modifies the saturation term [25]. Thus, the model is specified by:

(4) $$\begin{array}{cc} \frac{dM}{dt} & =(\alpha_{1}-\alpha_{2}-\alpha_{3})M \end{array}$$

(5) $$\begin{array}{cc} \frac{dE}{dt} & =\alpha_{2}M+\left(\beta_{1}-\beta_{2}-\beta_{3}-\frac{kE}{1+mE}\right)E, \end{array}$$

where the parameters are described in Table 1.

**Table 1:** The parameters that describe the linear and saturated feedback model. The values given are those used for the simulation of the saturated feedback model. The units of all of the *α* and *β* are [1/div], where div is the time for one cell division in days. The unit of *k* is [1/Ndiv], and the unit of *m* is [1/N], where *N* is the number of cells of species *E* at time *t*. EMT, epithelial-mesenchymal transition.

Parameter	Value	Definition
$\alpha_{1}$	0.32	Proliferative capacity of *M*
$\alpha_{2}$	0.32	Differentiation of M→E
$\alpha_{3}$	0.02	Cell death of *M*
$\beta_{1}$	0.87	Proliferative capacity of *E*
$\beta_{2}$	0.25	Senescence of *E*
$\beta_{3}$	0.4	Cell death of *E*
$\beta_{4}$	0.0025	Rate of EMT
*k*	0.0026	Defines carrying capacity of *E*
*m*	0.0036	Defines maximum per-capita growth rate

Initially, cell death is not considered in either tumor cell subpopulation ($\alpha_{3}=\beta_{3}=0$), the same situation that we later apply to a stochastic model. Furthermore, we assume that only asymmetric cell divisions occur for *M*; then, $\alpha_{1}=\alpha_{2}$, and we observe homeostatic conditions: $\frac{dM}{dt}=0$. No change in the stem cell population size occurs; a single pioneer stem cell remains the sole stem cell in the tumor cell population. Under these conditions, *M* acts as an effective parameter in Equation (2). When we introduce the effect of EMT on the system, we will have $\frac{dM}{dt}\neq 0$. In Figure 1, trajectories of *M* and *E* are shown for the two forms of the model. Refinement of the model to include cell death in *E* alters the population equilibrium and the trajectory to which the populations arrive at the equilibrium, but the system remains one where a stable population equilibrium persists. Similarly, altering the rate of proliferation in *M* and *E* or the number of cell divisions that *E* can undergo prior to senescence has similar effects; the trajectory of the population dynamics is altered, but the system still arrives at a stable equilibrium dynamic. In short, alterations in cell death, proliferation or the capacity of cells to continue to divide does not fundamentally alter the growth dynamics of the tumor cell populations beyond forcing the system to find a new stable equilibrium. Unless these parameters are dramatically shifted, likely beyond what might be physiologically plausible, it appears they might not be sufficient to allow metastatic tumors to exit dormancy. All simulations and sensitivity analysis of the ODE models were performed in MATLAB (R2013a; The MathWorks, Natick, MA, USA).

### 2.2. EMT Dramatically Alters Population Dynamics in the Deterministic Model

**Figure 2:**
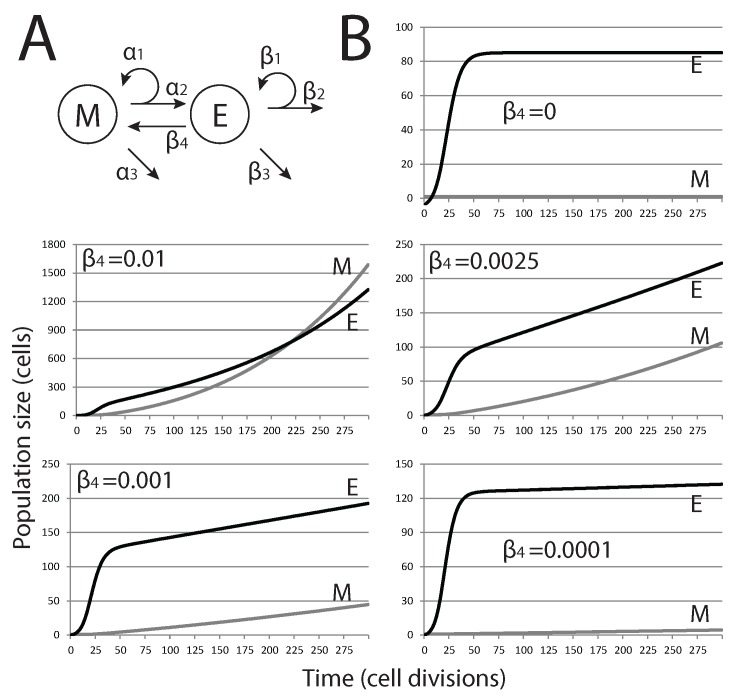
Effect of EMT on the size of metastatic tumors. (**A**) Modified model diagram, including EMT; (**B**) each plot shows the trajectories of *M* and *E* for different values of $\beta_{4}$ (rate of EMT). As $\beta_{4}$ is increased, the dynamics change from close-to-equilibrium to exponential growth, where eventually, the size of *M* will overtake *E*.

After defining the model under constraints outlined in the previous section, we then add EMT through an additional term to examine how it affects tumor dormancy (Figure 2A). The equations for the saturated feedback model thus become:

(6) $$\begin{array}{cc} \frac{dM}{dt} & =\left(\alpha_{1}-\alpha_{2}-\alpha_{3}\right)M+\beta_{4}E \end{array}$$

(7) $$\begin{array}{cc} \frac{dE}{dt} & =\alpha_{2}M+\left(\beta_{1}-\beta_{2}-\beta_{3}-\beta_{4}-\frac{kE}{1+mE}\right)E. \end{array}$$

The parameters are as above, and the values used for simulations are also given in Table 1. The parameter values chosen reflect the physiological conditions that we expect to observe in a clinical scenario. Given data on cell doubling times in cancer cell lines, we can set bounds on the values that parameters should take and, thus, ensure our model fits closely with the biological systems in question. The range of cell doubling times, as reported by Stinson *et al.* [34], is from 0.725–3.31 days (17.4–79.5 h); thus, we constrain proliferation rates to lie within the range of 0.302–1.38 cell divisions per day. For the simulation, we set $\alpha_{1}$, the proliferation rate of *M*, to $\alpha_{1}=0.32$, at the lower end of this range, since we expect stem-like cells to cycle slowly. We set $\beta_{1}$, the proliferation rate of *E*, to $\beta_{1}=0.87$, since we expect the epithelial population to have a faster rate of turnover. For the remaining parameters, where estimates from the literature were not available, we estimated values within the same order of magnitude as ($\alpha_{1},\beta_{1}$) and then studied them further within these ranges, as we describe below.

We first explored how variations in epithelial cell senescence and cell death (parameters $\beta_{2}$ and $\beta_{3}$, respectively) affected our model in conditions where EMT does no occur ($\beta_{4}=0$). Simulations show that populations grow from a single mesenchymal cell to reach a stable steady-state population. Changing senescence and cell death alters the steady-state population and how quickly this steady state is achieved. We therefore chose parameter values that would maintain a small micrometastatic tumor in a dormant steady state for further consideration.

As shown in Figure 2, when EMT is present ($\beta_{4}>0$), our model predicts that no steady state is reached, at least for conditions where mesenchymal cell death is ignored ($\alpha_{3}=0$). With the highest rates tested, exponential growth of both cell populations is observed, and the size of the mesenchymal stem cell population eventually exceeds that of the differentiated epithelial cell population. For lower, and likely more biologically relevant, rates of EMT, the size of the mesenchymal population is brought below that of the epithelial population, and the overall growth curve changes shape from exponential to nearly linear. Nevertheless, these tumors do not reach a steady state and grow continuously.

We then study how mesenchymal cell death ($\alpha_{3}>0$) affects the population dynamics when EMT occurs at a rate where tumor growth is rapid, but where mesenchymal and epithelial population numbers do not intersect. Reflecting the ability of stem cells to remain viable for many generations and reducing their susceptibility to undergo apoptosis, we only considered small values of $\alpha_{3}$. With cell death limiting population growth in *M*, the population dynamics are no longer continuous and arrive at some stable equilibrium state. Thus, colonization of a distant site by a single metastatic cancer stem cell can result in the formation of a small metastatic tumor that reaches maximum size and contains a specific ratio of mesenchymal cells and differentiated epithelial cells, as long as EMT and cell death in the mesenchymal cell population are balanced. With a very small number of cells at steady state, this could reflect the dormant state of a micrometastatic tumor. Changes in these parameter values, whether driven by mutation or changes in the tumor microenvironment, would dramatically alter the population dynamics in the tumor in a way that allows uncontrolled tumor growth.

With a model system that maintains a stable steady-state population with a small number of tumor cells, we employ local sensitivity analysis of each model parameter to determine which parameter values have the largest impact on tumor population dynamics. Here, altering values for parameters used for the simulation above allows us to quantify the relative effects of small changes in parameters on model output. We perturb each parameter independently by 5% and then calculate the sensitivity coefficient $\frac{\partial M}{\partial p}$, the change in the species *M* once the system has reached steady state (t=500), relative to parameter *p*. We find that the relative effect of EMT ($\beta_{4}$) is largest; the population dynamics are dramatically altered with small changes in this parameter value. For all other parameters, including cell proliferation and death rates, little effect is seen on the steady-state population abundances of *M* and *E*. The exception is $\alpha_{1}$, which has a moderate effect. (We define parameters with a significant effect on those with sensitivity coefficients greater than 10,000; described in Table 2.) In short, EMT events have the most dramatic effect on tumor population dynamics, and changes in their probability are the most likely to drive population dynamics away from a steady-state equilibrium (dormancy) and into continuous growth.

**Table 2:** Sensitivity in model output (steady-state level of *M*) to changes in parameters by ±5%. We see that the parameters with the greatest effect on output are $\beta_{4}$ (the rate of EMT) and $\alpha_{1}$ (the proliferative capacity of *M*).

Parameter (*p*)	Sensitivity Coefficient ($\frac{\partial M}{\partial p}$)
$\alpha_{1}$	10,200
$\alpha_{2}$	1,840
$\alpha_{3}$	3,260
$\beta_{1}$	449
$\beta_{2}$	355
$\beta_{3}$	358
$\beta_{4}$	29,700

### 2.3. Modeling Chemotherapeutic Treatment of Dormant Tumors

We use the saturated feedback model to study the effects of chemotherapeutic treatments on dormant metastatic tumors. The steady-state concentrations of *E* and *M* can be obtained by setting the left-hand sides of equations to zero and solving for *E* and *M*. There are two sets of solutions. One set of solutions is the non-zero (stable tumor population state), which is described mathematically by:

(8) $$\begin{array}{cc} E^{*} & =\frac{\beta_{2}\alpha_{3}+\beta_{3}\alpha_{3}+\beta_{4}\alpha_{3}-\alpha_{2}\beta_{4}-\beta_{1}\alpha_{3}}{\alpha_{2}\beta_{4}m+\beta_{1}m\alpha_{3}-\beta_{2}m\alpha_{3}-\beta_{3}m\alpha_{3}-\beta_{4}m\alpha_{3}-k\alpha_{3}} \end{array}$$

(9) $$\begin{array}{cc} M^{*} & =\frac{\beta_{4}\left(\beta_{2}\alpha_{3}+\beta_{3}\alpha_{3}+\beta_{4}\alpha_{3}-\alpha_{2}\beta_{4}-\beta_{1}\alpha_{3}\right)}{\alpha_{3}\left(\alpha_{2}\beta_{4}m+\beta_{1}m\alpha_{3}-\beta_{2}m\alpha_{3}-\beta_{3}m\alpha_{3}-\beta_{4}m\alpha_{3}-k\alpha_{3}\right)} \end{array}$$

whereas the other state that describes a tumor-free state is simply:

(10) $$\begin{array}{cc} E^{*} & =0,M^{*}=0 \end{array}$$

The model parameters chosen ensure that the analytical expressions of the two solutions above are non-negative and biologically plausible.

**Figure 3:**
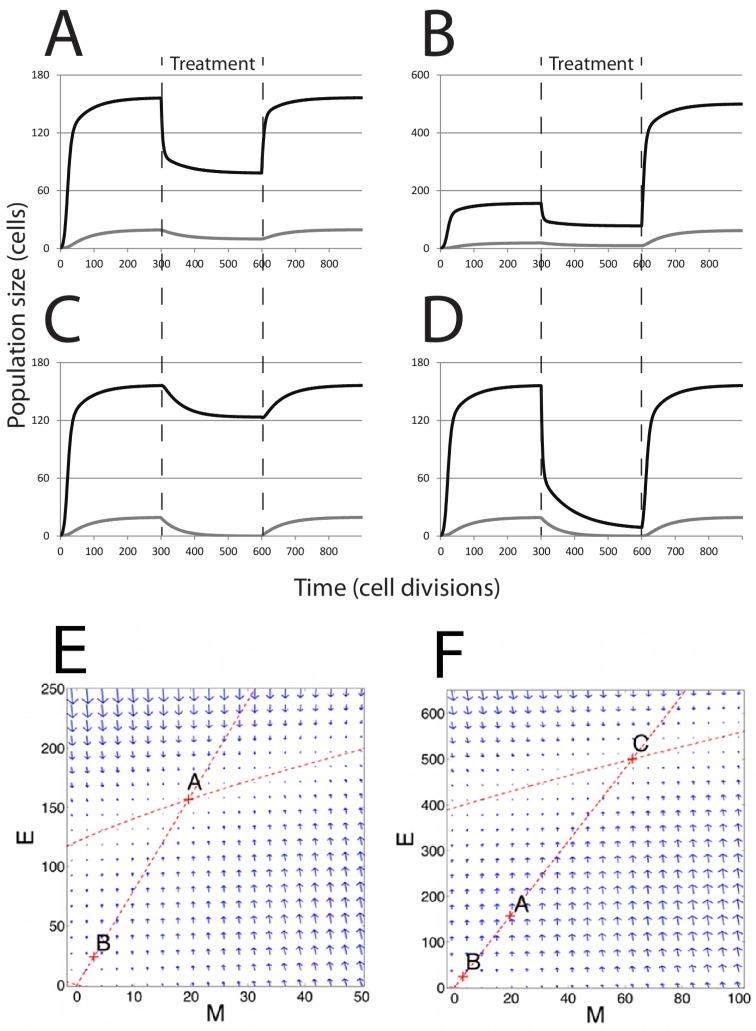
Population dynamics in response to chemotherapy. Modeling chemotherapeutic treatment and recurrence; tumor population dynamics before, during and after a treatment regime (denoted by the dashed vertical lines). (**A**) Treatment consisting of an agent that increased cell death ($\beta_{3}$) in the *E* cell population. At the end of treatment, $\beta_{3}$ returns to its pre-treatment level; (**B**) The *E* cell population acquires resistance mutations following treatment ($\beta_{3}$ is lower than before treatment); (**C**) Treatment consisting of an agent that prevents EMT; (**D**) Treatment consisting of both $\beta_{3}$ reduction and EMT prevention in combined therapy. In (C) and (D), recovery was to original pre-treatment parameter values; (**E**) The phase plane plot shows the behavior of the system around the fixed Point A (pre-treatment parameter values) and nullclines. Point B marks the system state after treatment; (**F**) Phase plane plot around the fixed Point C; parameter values after *E* cells have acquired mutations. In both (E) and (F), the fixed points shown are the only stable fixed points for the system in this state.

In the absence of chemotherapy treatment, any initial number of cells (E,M) will have trajectories that approach the solution given by Equations (8) and (9), indicated by point *A* in the phase plane (Figure 3E). By performing stability analysis under this parameter combination, Solution A is the only stable solution, whereas the tumor-free solution (Equation (10)) is always unstable, meaning that the system is unable to remain in the tumor-free state.

For our purposes, we model the effects of a chemotherapeutic treatment that increases cell death. First, we consider a treatment that only affects *E* cell death,* i.e.*, we increase $\beta_{3}$ by 33%, while *M* cell death is unaffected. The rationale for how we consider this treatment is based on the observation that mesenchymal cells are frequently highly resistant to chemotherapeutic agents [12]. The result is decay in both the mesenchymal and epithelial populations (Figure 3A,B) towards new steady-state values; the system moves from Point A to Point B in Figure 3E. However, after treatment ends, the parameters revert to their pre-treatment values, and the system returns to the same steady-state solution as before treatment, returning to Point A in Figure 3E. This would be true wherever the post-treatment Point B was located, unless total eradication of all cells was achieved. If the tumor cell population goes through a selective pressure that alters the proliferation or cell death parameters, the system will now move to a new steady state. This is modeled in Figure 3B,F, where $\beta_{3}$ is reduced below its initial, pre-treatment value, an effect that is meant to mimic acquisition of resistance to cell death following selection by the treatment regimen. Here, still, only one stable fixed point exists for the post-treatment tumor (now Point C); regardless of the starting values of (M,E), including Points A and B, the system will return to Point C.

We also tested the effect of a novel chemotherapeutic agent that prevents EMT ($\beta_{4}=0$). The result of this treatment, in isolation, is a decline in *M* and a much less pronounced decline in *E* (Figure 3C). Returning parameter values to prior levels (reflecting the end of the treatment regime) results in the tumor population returning to the prior steady state. Combined treatment was simulated by slightly increasing $\beta_{3}$ and reducing $\beta_{4}$ to zero simultaneously (Figure 3D). The result is an increased rate of population decline compared to either single therapy. Notably, the entire mesenchymal stem cell population is eliminated, and only a small number of differentiated cells remain. However, with even a single epithelial cell remaining and the ability to undergo EMT restored following cessation of drug treatment, the entire tumor is restored upon cessation of the treatment regime under this model.

**Figure 4:**
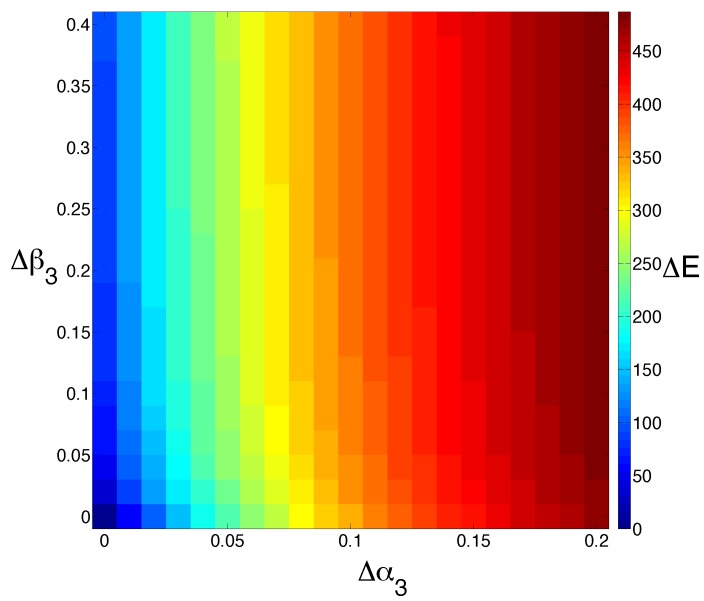
Model sensitivity to changes in cell death. We study the effect of increasing the cell death rate of *M* ($\alpha_{3}$) and *E* ($\beta_{3}$) simultaneously. The output is colored by the extent to which the steady state for *E* is shifted (ΔE). Increasing the rate of *M* cell death has a much more dramatic effect than increasing the rate of *E* cell death alone.

Since the assumption that *M* is completely resistant to chemotherapy may be unrealistic, we now look at the effects of changing *M* and *E* cell death rates simultaneously (Figure 4). Interestingly, we see that increasing *M* cell death $\alpha_{3}$ has more of a dramatic effect on the steady-state population size of *E* than increasing *E* cell death $\beta_{3}$.

At the parameter values chosen, the system never arrives at a tumor-free steady state, and regrowth of the tumor from the remaining cells is inevitable. We next ask whether a chemotherapeutic treatment affecting epithelial cell death ($\beta_{3}$) could totally eradicate tumor cells in this system. We find that a transition from a stable tumor population solution to a tumor-free solution can occur at a threshold value of $\beta_{3}$. This threshold can be determined by:

(11) $$\begin{array}{cc} \beta^{*} & =\beta_{4}\frac{\alpha_{2}}{\alpha_{3}}+\left(\beta_{1}-\beta_{2}-\beta_{4}\right) \end{array}$$

**Figure 5:**
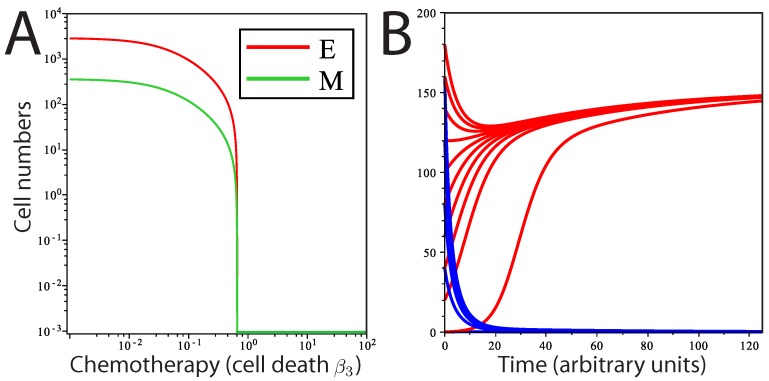
Transition from tumor to tumor-free state by induction of cell death. (**A**) Theoretical dose response curve of stable solutions of *E* and *M* as $\beta_{3}$ is varied; (**B**) solution curves of *E* at two different rates of cell death (by chemotherapy) with different initial numbers of cells. Red curves represent the solution of *E* for $\beta_{3}<\beta^{*}$; blue curves represent the solution of *E* for $\beta_{3}>\beta^{*}$.

The first term on the right-hand side describes the extent to which *M* contributes to the production of *E*, and the second term, $\left(\beta_{1}-\beta_{2}-\beta_{4}\right)$, describes the net growth of *E*. When $\beta_{3}=\beta^{*}$, the two solutions (tumor-free state and tumor state) intersect in a so-called transcritical bifurcation and exchange stability (Figure 5A). For $\beta_{3}<\beta^{*}$, all solutions converge to a stable tumor population (Equations (8) and (9) and Point A in Figure 3E), and the tumor-free state is unstable (Equation (10)). When $\beta_{3}>\beta^{*}$, then the population converges on a stable tumor-free state, while the tumor population solution has now become unstable. The model predicts that over time, when the level of cell death (due to chemotherapy) is above $\beta^{*}$ and regardless of the initial population levels, the concentrations of *E* and *M* will approach a stable tumor-free state, whereas below $\beta^{*}$, the system plateaus at a stable tumor population, regardless of the starting population size (Figure 5B). Consistent with our model, there is no bifurcation for any value of $\beta_{4}$ greater than or equal to zero, suggesting that variations in the rate of EMT in the tumor population cannot independently drive the system to a tumor-free state.

### 2.4. Defining a Stochastic Model of Dormant Metastatic Tumors

**Figure 6:**
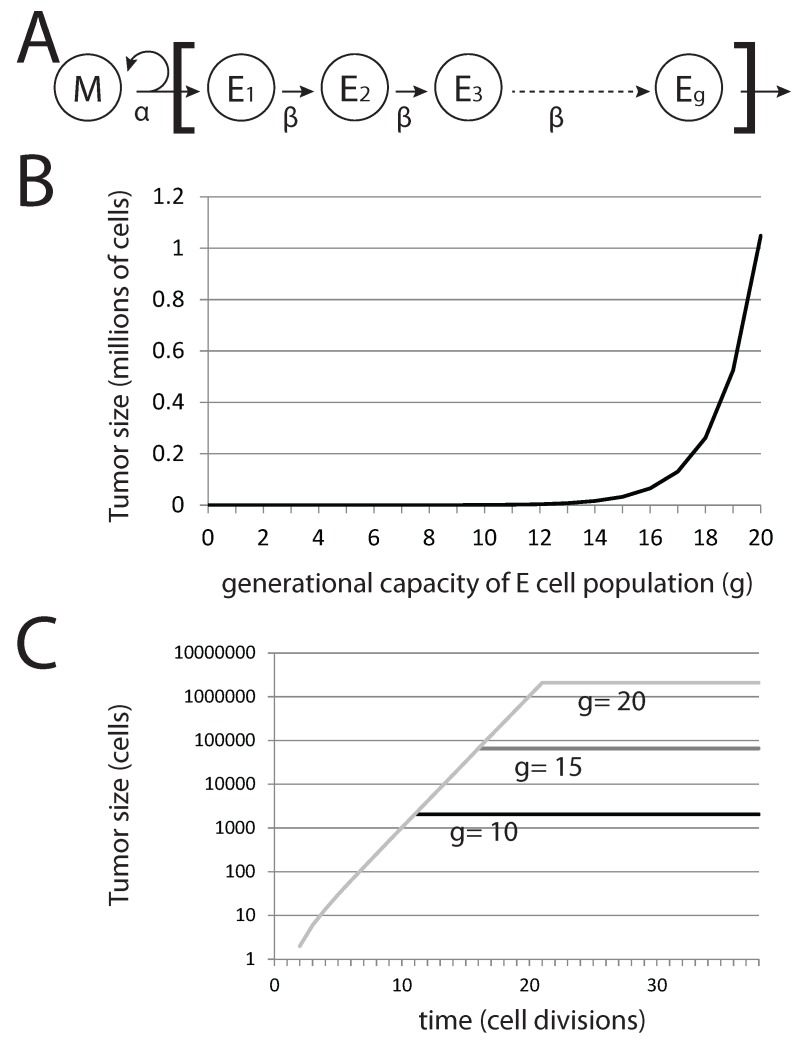
Generational capacity affects dormant tumor steady-state population sizes in a stochastic model. (**A**) The diagram represents the basic model, where mesenchymal stem cells *M* divide asymmetrically to generate differentiated epithelial cells *E* with a generational capacity (*g*) that measures the number of possible cell divisions that these cells can undergo prior to senescence; (**B**) steady-state population of tumors derived from a single pioneering *M* cell with varying *g*; (**C**) steady-state population sizes over time for tumors derived from a single *M* cell.

Given that deterministic models may not accurately reflect the behavior of very small tumor cell populations, where rates substitute poorly for probabilities, we develop a simple stochastic model for tumor population dynamics (Figure 6A). A stochastic description of metastasis allows us to study the frequency of tumor recurrence, rather than the rate of recovery. We produce a stochastic model in Python (Python Software Foundation, version 2.7, available at https://www.python.org), where a single pioneering cancer stem cell divides asymmetrically, generating a stem and a differentiated progeny cell at each division. While the stem cell can repeat such divisions indefinitely, the resulting differentiated epithelial cell can proliferate for a fixed number of generations, after which the daughter cells become senescent; we refer to this as the generational capacity.

In this basic model, the tumor population following metastatic colonization can be easily calculated. Without EMT, the total tumor population rapidly reaches a maximum value, after which it remains constant. The total tumor size *T* is given by:

(12) $$\begin{array}{cc} T & =2^{g}\left(1+N_{E}\right) \end{array}$$

where *g* is the generational capacity of epithelial cells and $N_{E}$ denotes the number of EMT events occurring in the tumor. Thus, variation in *g* (that is, the capacity of epithelial cells to undergo rounds of cell division before becoming senescent) affects the steady-state tumor population size and not the rate of growth prior to achieving this steady-state population level (Figure 6B,C). For the case in which no EMT events occur, we can immediately derive tumor size from *g* alone. Interestingly, for g=20, T≈1 million cells ($2^{20}=1,048,576$), a very small and perhaps undetectable final tumor volume is achieved. Reducing *g* generates tumors that are significantly well below detectable size. Thus, the presence of undetectable metastases can be explained by a tumor cell population that is founded by a single cancer stem cell and in which differentiated cells have a generational capacity of less than 20.

Importantly in this model, cells are lost only by senescence and not by cell death. Factoring in death by differentiated cells reduces the steady-state population size of this tumor cell subpopulation, as well as the rate at which the steady state is reached. Death of the single pioneering cancer stem cell in this model would result in the rapid decline and disappearance of the tumor cell population, as differentiated cells cease to be produced by the pioneering cancer stem cell. The result is a model where a small and highly stable tumor cell population is achieved from a single progenitor cancer stem cell, the model we use for dormant metastatic tumors.

### 2.5. A Stochastic Model of EMT for Small Differentiated Cell Populations

**Figure 7:**
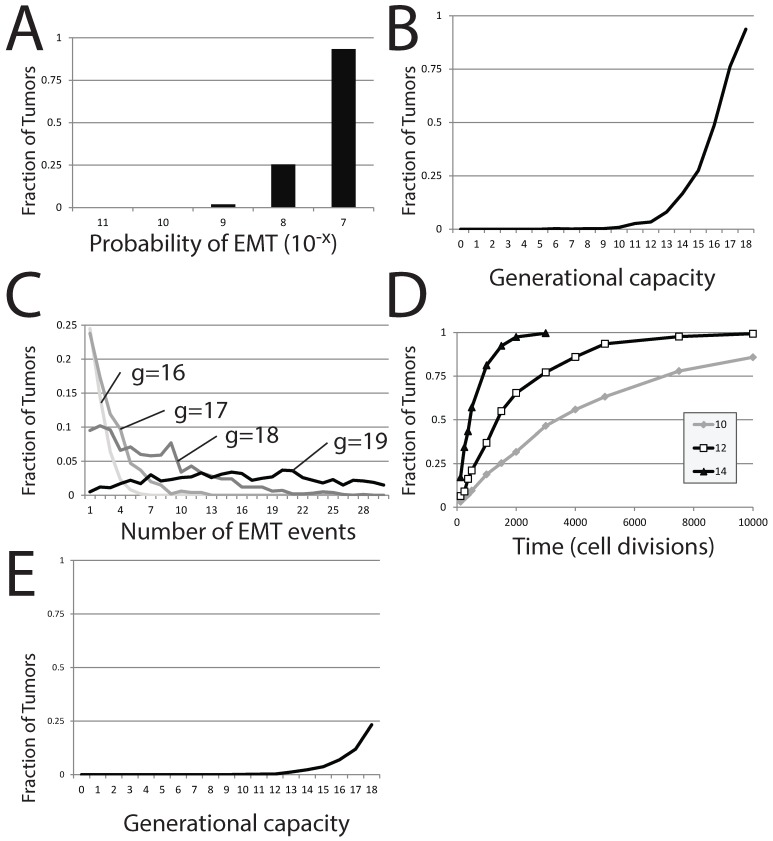
EMT in a stochastic model of dormant tumor recurrence. (**A**) The fraction of tumors in which EMT events occur with a fixed generational capacity of 18 and for different probabilities of EMT; (**B**) the fraction of tumors in which EMT events occur with a varied generational capacity and fixed probability of EMT; (**C**) the fraction of tumors exhibiting specific numbers of EMT events as generational capacity is altered; (**D**) the fraction of tumors exhibiting occurrences of EMT over time, for three different generational capacities; (**E**) the fraction of tumors in which EMT events occur with a varied generational capacity of 18 and fixed probability of EMT. In (**A**–**C**), tumors were initiated from a single precursor *M* cell; while in (**D**), tumors were started from a population of 100 *E* cells. The probability of EMT in (**B**–**E**) was set at $10^{-7}$.

We now consider the case where EMT occurs ($N_{E}>0$ in Equation (12)) in the dormant tumor cell population (Figure 7). We see tumor size *T* increase by a factor $\left(1+N_{E}\right)$. Clearly, recurrent EMT events within metastatic tumors will greatly alter tumor size; understanding how the frequency of EMT events might alter the tumor dynamics is critical to understanding metastatic tumor progression. We therefore develop a probabilistic description of EMT occurrences. We define *δ* to be the probability of an EMT event occurring in the E cell population during each cell division, analogous to the deterministic rate of EMT, $\beta_{4}$, modeled previously. We study the occurrence of EMT events as a function of tumor size and time. For each condition, we perform batches of 1000 individual simulations and track the number of individual simulations in which one or more EMT events occurs (Figure 7A). For fixed *g*, the occurrence of EMT events within individual simulations appears at a threshold probability. We vary *δ* in computation attempts where tumors are derived from a single stem cell (the differentiated cell population can undergo 18 subsequent divisions before senescence) and where the tumor is tracked for 120 cell generations in total. EMT events occur within a small fraction of the 1,000 individual simulations (1.9%) when $\delta =10^{-9}$. The number of simulations in which EMT events occur reaches 25% upon increasing *δ* to $10^{-8}$ and approaches 100% for even greater values ($\delta =10^{-7}$).

The generational capacity of the differentiated cell population also greatly affects the frequency of individual simulations demonstrating one or more EMT events (Figure 7B). Further, the total number of EMT events within each individual simulation also rises markedly with increases in *δ*; more EMT events occur in more individual simulations as generational capacity is increased (Figure 7C). However, the effect of generational capacity on the likelihood of EMT events in batches of simulations is partially an artifact of fixing analysis to a restricted period of 120 total cell generations. No matter the value of *g*, the fraction of trials in which one or more EMT events occurs approaches one as the time of analysis is increased, though the approach becomes more protracted as *g* is reduced (Figure 7D).

### 2.6. Dormant Tumor Recurrence in the Stochastic Model

We next use this stochastic model to assess the likelihood that a dormant metastatic tumor can recover from chemotherapeutic treatment that has successfully eliminated the entire *M* cell population and leaves only a small population of *E*. This scenario was the result of a largely successful chemotherapeutic campaign in our deterministic model. Using our stochastic model, we perform simulations that start with no mesenchymal cells and a small number of epithelial cells, E=10. In this scenario, recovery of the tumor occurs if an EMT event occurs before the entire population of *E* is lost to senescence. In batches of these trials, the generational capacity of the differentiated cells is varied, but it is assumed that the entire starting population retained its full generational capacity, despite this worst case scenario being unlikely. We count the number of individual simulations within the entire batch showing at least one EMT event, as this would result in reestablishment of the dormant tumor, at a minimum. As expected, the effect is strikingly similar to results started from a single *M* cell; increasing *g* dramatically increases the proportion of individual trials in which EMT events occur (Figure 7E), though the number of trials displaying one or more EMT events is much lower. A generational capacity of 18 gives rise to individual simulations with one or more instances of EMT in 233 of 1000 trials. The reduction in the proportion of trials showing EMT as *g* is reduced is dramatic; 11.9% of trials exhibit EMT events when *g* is 17, 6.9% when *g* is 16, 3.7% when *g* is 15 and below 2% when *g* is lower than 14. While small numbers of *E* cells can recapitulate the entire tumor, this occurs in a diminishing number of cases as the generational capacity of tumor cells is reduced.

## 3. Discussion

Dormant metastatic tumors, established from invasive cells that detach from the primary tumor, dramatically affect long-term patient survival. Colonizing cells that establish dormant tumors are thought to exhibit stem cell-like properties that can reconstitute a rapidly growing tumor upon exiting the dormant state. Maintenance of dormancy is not well understood, though widely suspected mechanisms include cell cycle arrest, insufficient vascularization and immune response suppression. Here, we employ a mathematical model of micrometastatic tumors that exist in a dormant state resulting from balanced proliferation and cell death. We use this general and simple model to assess how specific cellular behaviors contribute to the tumor’s exit from the dormant state, including the contribution of epithelial-mesenchymal transition (EMT) events. Our goal is to understand the general principles of tumor dormancy as they might apply across a number of cancer types.

In our dormant tumor model, a balanced proliferation model adapted from Johnston *et al.* [25], colonizing mesenchymal cells that bear stem cell properties divide asymmetrically to yield a single mesenchymal cell and a single differentiated epithelial cell with each division. Differentiated epithelial cells can undergo a limited number of divisions before becoming permanently senescent. This results in tumors with stable cancer cell populations, the size of which is largely affected by what we term generational capacity (the ability of differentiated cells to undergo repeated cell divisions before becoming senescent) and by the balance of proliferation and cell death. Since this model yields a highly stable population equilibrium, it recapitulates the balanced proliferation model of tumor dormancy [30]. A high death rate in the differentiated cell population, a result of a tissue environment that is not well suited to the differentiated cell population, could maintain a low equilibrium tumor size with very few cells.

We find that alterations in parameters affecting proliferation and survival do affect population dynamics in dormant tumors, but do so in such a way that a new equilibrium state is achieved without generating continuous tumor growth. In short, increasing the rate of proliferation, decreasing survival or altering the generational capacity of differentiated cells allows the tumor to reach a larger size at equilibrium, but does not result in continued tumor growth beyond this equilibrium. In contrast, we find that altering the rate of EMT, namely the ability of epithelial cells to revert to a mesenchymal cell type before becoming senescent, dramatically affects population dynamics in such a way that continuous growth can be achieved with relatively small changes in the parameter value. Varying this parameter could result from a stochastic event (such as the acquisition of a random mutation) or a change in the tumor microenvironment, either of which provide a biological explanation for altering the probability of observing EMT.

Modeling the chemotherapeutic treatment of dormant micrometastatic lesions is also informative. A drug that targets the differentiated epithelial cell population (increasing epithelial cell death) drastically reduces the population size. Drugs that prevent EMT do little alone; but good treatment results are achieved when EMT prevention is combined with traditional therapy. Here, the population of mesenchymal cell is reduced to zero, and differentiated cells are almost eradicated, though some differentiated cells remain. In our model, a single mesenchymal cell will recapitulate the dormant tumor upon cessation of therapy. Further, eradication of the mesenchymal cell population is not sufficient to prevent recurrence, as mesenchymal cells can be generated if an EMT event occurs within the residual differentiated epithelial cell population. Our stochastic modeling of tumor recovery from EMT events occurring in small populations of residual differentiated epithelial cells following the end of treatment shows that the probability of tumor recovery can be quite high, presenting a challenge to developing strategies for the eradication of dormant tumors. Continued treatment with an EMT inhibitor until the last differentiated cell reaches senescence is one obvious possibility.

Though we have not directly addressed mutations that confer an unlimited generational capacity to the differentiated epithelial cell population, the results are nonetheless clear: the probability of EMT, and, thus, of tumor recurrence, becomes certain if differentiated cell possess an unlimited generational capacity. Conversely, any treatment that would reduce the generational capacity of the differentiated cells would dramatically improve outcomes in attempts to eradicate dormant micrometastatic tumors.

Dormant metastatic tumors greatly complicate treatment, but the underlying biology of dormancy remains poorly defined. The underlying biology is complex and likely to behave in unpredictable ways, making the generation of hypotheses based on intuitive understanding likely to be unsuccessful. When experiments are technically challenging or consume significant resources, as is the case for tumor dormancy, modeling emerges as an important tool that allows for the refinement of hypotheses without a reliance on intuition. Modeling can thus be key to identifying critical experiments that are most likely to prove fruitful in improving our understanding. Our data suggest that the frequency of EMT events may be the critical driver for determining whether micrometastatic tumors exit dormancy. Experimental efforts to test this idea could be very important to progress in the field.

## Abbreviations

EMT, epithelial-mesenchymal transition; ODE, ordinary differential equation.

## References

[1] Fidler I.J. (2005). Cancer biology is the foundation for therapy. Cancer Biol. Ther..

[2] Uhr J.W., Pantel K. (2011). Controversies in clinical cancer dormancy. Proc. Natl. Acad. Sci. USA.

[3] Croker A.K., Allan A.L. (2007). Cancer stem cells: Implications for the progression and treatment of metastatic disease. J. Cell. Mol. Med..

[4] Hermann P.C., Huber S.L., Herrler T., Aicher A., Ellwart J.W., Guba M., Bruns C.J., Heeschen C. (2007). Distinct populations of cancer stem cells determine tumor growth and metastatic activity in human pancreatic cancer. Cell Stem Cell.

[5] Fiala S. (1968). The cancer cell as a stem cell unable to differentiate. A theory of carcinogenesis. Neoplasma.

[6] Todaro M., Perez Alea M., Scopelliti A., Medema J.P., Stassi G. (2008). IL-4-mediated drug resistance in colon cancer stem cells. Cell Cycle.

[7] Eramo A., Ricci-Vitiani L., Zeuner A., Pallini R., Lotti F., Sette G., Pilozzi E., Larocca L.M., Peschle C., de Maria R. (2006). Chemotherapy resistance of glioblastoma stem cells. Cell Death Differ..

[8] Johnston M.D., Maini P.K., Chapman S.J., Edwards C.M., Bodmer W.F. (2010). On the proportion of cancer stem cells in a tumour. J. Theor. Biol..

[9] Dalerba P., Cho R.W., Clarke M.F. (2007). Cancer stem cells: Models and concepts. Ann. Rev. Med..

[10] Radisky D.C., LaBarge M.A. (2008). Epithelial-mesenchymal transition and the stem cell phenotype. Cell Stem Cell.

[11] Polyak K., Weinberg R.A. (2009). Transitions between epithelial and mesenchymal states: Acquisition of malignant and stem cell traits. Nat. Rev. Cancer.

[12] Arumugam T., Ramachandran V., Fournier K.F., Wang H., Marquis L., Abbruzzese J.L., Gallick G.E., Logsdon C.D., McConkey D.J., Choi W. (2009). Epithelial to mesenchymal transition contributes to drug resistance in pancreatic cancer. Cancer Res..

[13] Scheel C., Weinberg R.A. (2012). Cancer stem cells and epithelial-mesenchymal transition: Concepts and molecular links. Semin. Cancer Biol..

[14] Yang J., Weinberg R.A. (2008). Epithelial-mesenchymal transition: At the crossroads of development and tumor metastasis. Dev. Cell.

[15] Thiery J.P. (2003). Epithelial-mesenchymal transitions in development and pathologies. Curr. Opin. Cell Biol..

[16] Day R.M., Felici A., Bottaro D.P., Savagner P. (2005). Hepatocyte growth factor regulates transitions between epithelial and mesenchymal cellular phenotypes during normal development and in disease. Rise and Fall of Epithelial Phenotype Concepts of Epithelial-Mesenchymal Transition.

[17] Naldini L., Vigna E., Narsimhan R.P., Gaudino G. (1991). Hepatocyte growth factor (HGF) stimulates the tyrosine kinase activity of the receptor encoded by the proto-oncogene *c-MET*. Oncogene.

[18] Weidner K.M., Sachs M., Birchmeier W. (1993). The Met receptor tyrosine kinase transduces motility, proliferation, and morphogenic signals of scatter factor/hepatocyte growth factor in epithelial cells. J. Cell Biol..

[19] Li Y., Li A., Glas M., Lal B., Ying M., Sang Y., Xia S., Trageser D., Guerrero-Cázares H., Eberhart C.G. (2011). c-Met signaling induces a reprogramming network and supports the glioblastoma stem-like phenotype. Proc. Natl. Acad. Sci. USA.

[20] Li C., Wu J.J., Hynes M., Dosch J., Sarkar B., Welling T.H., Pasca di Magliano M., Simeone D.M. (2011). c-Met is a marker of pancreatic cancer stem cells and therapeutic target. Gastroenterology.

[21] Jun H.J., Bronson R.T., Charest A. (2014). Inhibition of EGFR induces a c-MET-driven stem cell population in glioblastoma. Stem Cells.

[22] Tang M., Zhou H.Y., Yam J., Wong A. (2010). c-Met overexpression contributes to the acquired apoptotic resistance of nonadherent ovarian cancer cells through a cross talk mediated by phosphatidylinositol 3-kinase and extracellular signal-regulated kinase 1/2. Neoplasia.

[23] Scheel C., Weinberg R.A. (2012). Cancer stem cells and epithelial-mesenchymal transition: Concepts and molecular links. Semin. Cancer Biol..

[24] Patel P., Chen E.I. (2012). Cancer stem cells, tumor dormancy, and metastasis. Front. Endocrinol..

[25] Johnston M.D., Edwards C.M., Bodmer W.F., Maini P.K., Chapman S.J. (2007). Mathematical modeling of cell population dynamics in the colonic crypt and in colorectal cancer. Proc. Natl. Acad. Sci. USA.

[26] Anderson A.R.A., Enderling H., Chaplain M.A.J., Beheshti A., Hlatky L., Hahnfeldt P. (2009). Paradoxical dependencies of tumor dormancy and progression on basic cell kinetics. Cancer Res..

[27] Roeder I., Horn M., Glauche I., Hochhaus A., Mueller M.C., Loeffler M. (2006). Dynamic modeling of imatinib-treated chronic myeloid leukemia: Functional insights and clinical implications. Nat. Med..

[28] MacLean A.L., Lo Celso C., Stumpf M.P.H. (2013). Population dynamics of normal and leukaemia stem cells in the haematopoietic stem cell niche show distinct regimes where leukaemia will be controlled. J. R. Soc. Interface.

[29] MacLean A.L., Filippi S., Stumpf M.P.H. (2014). The ecology in the hematopoietic stem cell niche determines the clinical outcome in chronic myeloid leukemia. Proc. Natl. Acad. Sci. USA.

[30] Wells A., Griffith L., Wells J.Z., Taylor D.P. (2013). The dormancy dilemma: Quiescence versus balanced proliferation. Cancer Res..

[31] Manish P., Sylvia N., Sylvia N. (2006). Mathematical models of cancer. Cancer Bioinformatics: From Therapy Design to Treatment.

[32] Turner C., Kohandel M. (2010). Investigating the link between epithelial-mesenchymal transition and the cancer stem cell phenotype: A mathematical approach. J. Theor. Biol..

[33] Turner C., Kohandel M. (2012). Quantitative approaches to cancer stem cells and epithelial–mesenchymal transition. Semin. Cancer Biol..

[34] Stinson S.F., Alley M.C., Kopp W.C., Fiebig H.H., Mullendore L.A., Pittman A.F., Kenney S., Keller J., Boyd M.R. (1992). Morphological and immunocytochemical characteristics of human tumor cell lines for use in a disease-oriented anticancer drug screen. Anticancer Res..

